# Excerpta

**Published:** 1880-12

**Authors:** 


### EXCERPTA.

### A NEW ANTI-NEURALGIC: MENTHOL.

Archibald D. MacDonald, in a paper communicated to the Medico-Chirurgical Society of Edinburgh, by Dr. Taylor, Jan. 8, 1880, and published in the *Edinburgh Medical Journal*, Aug., 1880, urges the claims of menthol as a new antiseptic and anti-neuralgic agent. Menthol is the stearoptine of peppermint oil, is a crystalline solid derived from the oil of peppermint by long keeping, or being cooled to a low temperature. The American oil yields it at O°C. Its chief source is the Chinese and Japanese oil. It is imported as Japanese camphor, in small white crystals, which have the peppermint odor, and resembles the sulphate of magnesia. In liquid or solid state it has specific gravity less than that of water. It is rendered liquid and volatile at a temperature one or two degrees below that of the body. Some specimens, however, volatilize, at a much lower temperature. .It is very sparingly soluble in cold water. It liquifies slowly in water at 82° F., quickly at 120° F., but remains mostly as a separate layer. It dissolves freely in alcohol, in ether, in the fixed and volatile oils and in glycerine. The author publishes a series of experiments proving that this agent, which is the homologue of thymol, is an antiseptic of considerable power, but says that in Great Britain it will probably not come into general use as an antiseptic; but in India, China and Japan, and even in America, where the objection of small supply and high price cannot apply, its use is more to be maintained. One point is strongly urged in its favor, its volatilization at and a little below the temperature of the body, and its consequent adaptability for penetrating into every nook and cranny of wounds which are already petrefactive, slaying the bacteria, and rendering these wounds perfectly asceptic.

As an anti-neuralgic, the author believes the menthol to be of great value. One-tenth to one-sixth of a grain of menthol steadies the contractions of an excited heart, and slows them to a comfortable speed, and at the same time produces slight cerebral drowsiness. Half a grain of crystals produced vomiting from gastric irritation. The author does not advocate its internal use unless well diluted. He deals with menthol as a local anæsthetic. The application of oil of turpentine in facial neuralgia by the Chinese, was practiced at a very early date; it has also been locally applied for gout, the relief in both affections being almost instantaneous. A priori, the author concludes the oil cures in virtue of the menthol which it contains. Reference is made to seven cases. In the first case, tri-facial neuralgia, the affected part was painted with menthol, one grain; oil of clove, ten minims; rectified spirit, fifty minims. This was done four times in successive attacks. Entire relief followed in from four to six minutes. First a feeling of coldness, and then some irritation from the application was experienced. This case may be regarded as typical; menthol is particularly advocated as a temporizing remedy until constitutional treatment may take effect. It may prove to be more effective than aconite, veratria or atropia. Its use in toothache was followed by immediate relief. In neuralgia it has been noticed to render the paroxysms less frequent and less severe. In sciatica, the author painted the sciatic, popliteal and tibial nerves to the ankle joint with a ten per cent. solution, and this gave the patient immediate relief. —*Chicago Med. Journal.*

### SUBCUTANEOUS INJECTION OF ETHER IN SCIATICA.

Dr. Comegys recommends hypodermic injection of sulphuric ether for the treatment of sciatica (*L' Union Medicale*, August 5.) He cites two cases, one in detail, which he has cured by this plan. Three drops of ether are injected at intervals of twelve hours. The injection need not be a deep one; and, though it causes a momentary sharp pain, it does not bring any consecutive unpleasant effects. Dr. Comegys is inclined to think that the same injection might be successful in case of tic douloureux, for which Dr. Merino recommends hypodermic injection of ergotine.—*Canada Medical and Surgical Journal.*

### IODINÉ IN TYPHOID FEVER.

Prof. Roberts Bartholow recommends Tr. iodi. gtt. v., well diluted with water, in the treatment of typhoid fever. Under this—one of the German so-called specific plans of treatment—he says, "with proper diet and nursing, the mortality is much diminished." The same writer, for the diarrhœa of that disease, prefers the following: ℞ Liq. Potass. Arsenit. gtt. ii., Tr. Opii gtt. iv., repeated often as required.—*Canada Medical and Surgical Journal.*

### REMEDIES FOR HEADACHE.

The following recipes and suggestions for the treatment of different forms of headache are collected from a variety of trustworthy sources:—

Two grains of caffeine, in capsule, taken every half-hour, is a very effectual remedy in nervous and sick headache. One or two doses are often sufficient to give complete relief. The only objection to its use is sleeplessness, which sometimes results if it is taken in the evening. It is preferable to guarana as being hardly ever rejected by the stomach.

The following, according to Dr. W. W. Carpenter, is very effectual in most forms of headache:—

Muriate of ammonia, 3 drachms; acetate of morphia, 1 grain; citrate of caffeine, 30 grains; aromatic spirits of ammonia, 1 drachm; elixir of guarana, 4 ounces. Mix. Dessertspoonful every ten or twelve minutes.

In nervous headache, Dr. W. A. Hammond states the value of various drugs as follows:—

Oxide of zinc is of great value. Ordinary dose, 2 grains, three times a day, after meals; maximum dose, 5 grains. It is best given in form of pills.

Nux Vomica is preferable to strychnia. The dose is ¼ grain, after meals. If the patient be cholorotic, it is well to combine a grain of reduced iron and half a grain of sulphate of quinine.

Bismuth, in form of subcarbonate, will often take the place of oxide of zinc. Dose, 2 grains, after each meal. Bismuth probably aids digestion more than any mineral tonic, and is of use where there is gastric disturbance.

The bromides are serviceable when the nervous system has been irritated; when it is exhausted they do harm.

Phosphorus is very useful in most forms of nevous headache. The best results are obtained from dilute phosphoric acid, in doses of 30 drops, largely diluted, three times a day, after eating, or phosphite of zinc, 1-10 grain, in pill, three times a day.

Arsenic, as a nerve tonic stands next in value to zinc. Dose, 5 drops of Fowler's solution, three times a day, after meals.

Galvanism is sometimes valuable, but by no means a specific. The *constant current* should always be used, being careful to avoid too great intensity, less amaurosis be produced.

Dr. T. Lauder Brunton, editor of the London *Practitioner*, says: The administration of a brisk purgative, or small dose of epsom salts, three times a day, is a most effectual remedy for frontal headache when associated with constipation; but if the bowels be regular, the morbid processes on which it depends seem to be checked, and the headache, removed even more effectually, by nitro muriatic acid,diluted, 10 drops in a wineglass of water, or bicarb. soda, 10 grains, in water, before meals. If the headache be immediately above the eyebrows, the acid is best; but if it be a little higher up, just where the hair begins, the soda appears to be the most effectual. At the same time that the headache is removed, the feeling of sleepiness and weariness that frequently leads the patients to complain that they rise up more tired than they lie down, generally disappears.

A writer in the London *Lancet* remarks: At the Middlesex Hospital, female patients who have suffered many years from sick headache, evidently of a hereditary character, have been greatly benefited, if not cured, by the administration of 10 minim doses of tincture of Indian hemp, three times daily, between the attacks. This is well worthy of trial in those cases of ever-living, never-dying martyrdom-like suffering.

In headache due to determination of blood to the head and in fever, the following simple treatment is to be commended:—

Put a handful of salt into a quart of water, add an ounce of spirits of hartshorn and half an ounce of spirits of camphor. Cork the bottle tightly to prevent the escape of the spirit. Soak a piece of soft cloth with the mixture and apply it to the head; wet the rag fresh as soon as it gets heated.

Soaking the feet in very warm water, in which a spoonful of mustard has been stirred, is also beneficial in drawing the blood from the head.

Two teaspoonsful of powdered charcoal, well stirred in half a glass of water and drank at once, is a valuable remedy in sick headache from sour stomach, flatulence, etc.

Tincture of nux vomica is recommended by Ringer as possesed of real curative powers, when given in drop doses, repeated every 5 or

10 minutes for 8 or 10 doses, and then continued at longer intervals, for sick headache, accompanied with acute gastric catarrh, whether due to error in diet, constipation, or no apparent cause.—*Boston Journal of Chemistry*, Sept., 1880.

### CREOSOTE IN PYTHISIS.

The suggestion of creosote in phthisis is by no means new. Recently it became the subject of a discussion at one of the French medical societies, started by a paper by Dr. Boyer. In answer to inquiries, he replied that he directed his treatment mainly against the abundant expectoration, the profuse sweating, the diarrhœa, etc., which reduce the strength of the patients. Out of fifty patients who presented these symptoms, eight might be said to have been radically cured. The creosote appears to exercise a direct influence on the pulmonary phenomena, the primary cause of the above manifestations, and consequently the patients increase in weight in a manner regular and continuous. M. Grenet said he employed creosote where vomicæ existed, but did not obtain good results from it. Arsenic and cod-liver oil have, on the contrary, procured ameliorations which manifested themselves in certain patients by an increase of weight. He would ask M. Boyer why he objected to cod-liver oil, which proved its efficiency over and over again, and in what form of phthisis and in what dose he administered the creosote? M. Boyer, in replying, said he preferred creosote to arsenic and cod-liver oil, because the results appeared to him more constant and rapid. He had also recourse to glycerine combined with iodide of potassium, because this medicine is easily borne and accepted by the patients. In painting the thoracic walls with tincture of iodine, he considered that enough iodine was absorbed without having recourse to cod-liver oil. He administered the creosote as follows:

| | | |
|---|---|---|
| ℞ | Creosote, - - - | ʒ ij |
| | Rum, - - - | ℥ iv |
| | Glycerine, - - - | ℥ iv. |

One or two tablespoonfuls to be taken daily, after meals, in a little soda water.

It was in the torpid and scrofulous forms of phthisis arrived at the second stage that M. Boyer used the above mixture.—*Philadelphia Med. and Surg. Reporter.*

### BLOOD STAINS.

The startling thought having occurred to Dr. Chas. O. Curtman, of St. Louis, that there was a possibility of the transfer of human blood by predatory insects, such as mosquito, bed-bug, etc., he was led to make the following experiment: Mosquitoes were kept in close confinement after imbibing their fill of human blood. At different periods of time they were crushed, and the blood examined in various menstrua. In all cases, up to forty-eight hours after a meal, a large proportion of human blood corpuscles were unchanged and readily recognizable. The size and color of mosquito blood are very different from human. As the result of more than one hundred careful measurements, he gives the following sizes: Human blood (after imbibition by the mosquito), averages, in dilute glycerine, 1-3200 inch: in 80 per cent. alcohol, 1-4000 inch. Mosquito blood averages in dilute glycerine, 1-14000 inch: in 80 per cent. alcohol, 1-18000 inch. Dr. Curtman regrets that another prop is thus taken from the value of circumstantial evidence; for even if stains should be fully identified as derived from human blood, the accused may plead that they were due to the agency of insects. Later experiments prove that bed-bugs digest human blood far more readily than the mosquito; after twelve hours no trace of human blood being discovered. Verily, the criminal should dwell in the land of the mosquito. We suggest the wide publication of these investigations for the benefit of the public, criminal lawyers in particular.—*Louisville Medical Herald.* J. B. M.

### HOW THE METRIC SYSTEM HAS BEEN GETTING ON.

A calm survey of the field during the past summer leads to the conclusion that the extraordinary dash with which the metric system rushed into the arena a year or two ago, into this country, was a false start. A certain number of youthful doctors took it up with zeal, just as they do the new remedies which are boosted every month or two; a certain number of old doctors bored themselves greatly by learning and using it, for fear they would be called old fogies by their younger competitors. Several medical journals and one or two publication committees of State Societies passed decrees violently expelling those old friends the ℈, ʒ and ℥ from their pages. A huge reform was immediately to be inaugurated and carried by a sweeping vote.

A perceptible cooling off has followed within the last six months. The medical journals alluded to have quietly remitted the banished old friends on divers occasions; and the metric doctors, young and old, are shocked to discover that their impetuous welcome to the French stranger may possibly have been misplaced, and is not likely to be shared by their compatriots in the profession.

To show the justice of this opinion, we shall quote the words of various esteemed contemporaries, which will indicate conclusively, we take it, that the tide has turned, and that we had better drop the effort to force into prescription writing the method so hotly urged upon us.

The Detroit *Lancet* has put very strongly the fact that the meter is not an arbitrary, but a confessedly erroneous standard. It quotes with approval the expression of the eminent astronomer, John Hershel, to the effect, that the " production of the meter was not a blunder only, it was a sin against geometrical simplicity. It was a sin, because in the earth's axis they had a straight and unvarying line."

This is a knock-down blow from an authority which it were vain to question. It puts an end to the metrical system's claim to be based on an invariable and natural relation.

From the same quarter, the *Michigan Medical News* speaks in equally decided terms. In its issue of August 10th, we read:—

" The advocates of the metric system are very persistent in their efforts to foist it on the profession. In season and out of season they are advocating its claims, and urging that it supplant the system of weights and measures now in use. They have succeeded in achieving for it a foothold in the coming decennial pharmacopœia—a foothold which, unless care be exercised, will result in a repetition of the camel's intrusion, in the fable. There is no objection to its employment in pharmacy, that is, for the purpose of manufacturing, but to introduce it into the dispensing of drugs will be to cause great confusion.

"The simplicity of the metric is urged. This is an illusion. It admits of but one direction, and then a complexity begins, from which only a specially endowed mathematical mind can extricate itself, with any degree of certainty.

This last point has been well developed in the Atlanta *Medical Journal*, by Dr. C. W. Erwin. It is psychologically true, and the result of all experience, that the mind conceives more easily a quantity expressed in a few large units than in many small ones. The distinctions of coinage all recognize this. Why have dollars when one could speak of hundreds of cents; why cents when we could speak of thousands of mills, except for this obvious reason? Dr. Ennis' remarks are as follows:—

"No system should take precedence of the old that is not, 1st, as accurate; 2nd, as easily and quickly calculated; 3d, does not express weight, quantity, bulk, or measure; present to the mind as rapidly, and, as nearly as can be, the exact appreciation of the quantity, weight or measure expressed by the numbers.

It is not only necessary to calculate by arithmetic the vulgar or decimal fractional part of any unit of measure, but when the answer is called, the mind should grasp the idea of the actual quantity or bulk expessed by figures.

"Now we contend that the lower the numbers, and few the figures, the more readily the mind takes the idea. For this reason experience has taught us when figures expressing quantity, weight or measure become too numerous it is best to resort to lower figures of a higher denomination."

The *Western Lancet*, San Francisco, observes:

"It is an astonishing fact, that in looking over medical literature for a year or two back, we find so few objections to the system, if adopted into our tex-book, will render the reading ambiguous and tangling, unpracticed and unused; subjecting the entire text-book to the caprices of an unnatural regime."

The *Medical Annals*, Albany, in the June number, sets forth strongly the inaccuracy of the system and distinctly avowed their determination not to adopt it.

The *Medical Herald*, of Louisville, calls the whole system "an absurdity," and praised the Association of Medical Editors that they had declined to recommend it.

Meanwhile the American Medical Association, at its late meeting, recommended the adoption of this system, and is endeavoring to force its adoption by medical colleges, medical and pharmaceutical institutions and by medical men. Its committee is to report all medical colleges who do not adopt it.

Of this action Dr. Gaillard writes, in his *Medical Journal*:

"The whole effort will be an absolute failure. Nothing less than a law making its adoption imperative by the representatives of all avocations using weights and measures can secure its use by physicians. It is idle to use resolutions and reports for this purpose. It is courting failure.

The inconsiderate action of the American Medical Association has very naturally developed opposition. It is rather too much to have an awkward and erroneous system thus crammed down the throats of the profession, will they, nil they. This opposition has crystalized itsself into the form of an "International Institute for Preserving Weights and Measures." Branches of this Society have been organized in Boston, Cleveland, Pittsburg and elsewhere. Charles Latimer, of Cleveland, has written a pamphlet on "The French Metric System," published by Thos. Wilson, 188 Monroe St., Chicago, which claims goes to show that the time and place of the French Revolution and the worship of the Goddess of Reason did not produce a metric system worthy of adoption by the world.

For our own part, we have, as readers will remember never advocated the forcible introduction of the metric system into perscription writing. Societies and Journals travel widely beyond their proper spheres in trying to force their members and correspondents to use it. It is at best of questionable accuracy; it is more likely to lead to errors in compounding; it is less rational than the present method, and it is more difficult to comprehend the use. Its only real advantage is its decimal notation, and we can get that without resorting to it.—*Med. and Surg. Reporter.*

### ANTISEPTIC TREATMENT OF TYPHOID FEVER.

Dr. C. G. Rothe, after having treated 25 cases, recommends very highly a combination of Iodine and carbolic acid, viz., 1 to 2 of acid, and one of tincture of iodine, in 120 of water. All the patients, after the first days, as soon as the gastric symptoms had subsided, asserted they felt quite comfortable; and this subjective feeling lasted uninterruptedly to their convalescence. The latter also, in all, went on without disturbance, and without interruption by those troublesome slight relapses, which frequently seem to indicate, in the fourth or fifth week, some recrudescence of the local lesion. The medicine itself is readily taken by the patients, both children and adults; and, indeed for weeks; which cannot be said either of quinine or of salicylate of soda. Oil of peppermint completely disguises the disagreeable smell; and gastric or sensorial disturbances, which sometimes attend the use of the above-mentioned remedies were never observed. The medicine has also the recommendation of cheapness, a very important circumstance in view of the present high price of quinine. It seems important that the remedy should be given in sufficient quantities, a table spoonful being given hourly, until a decided effect on the pulse and temperature is produced, and then every two hours, until apyrexia follows; and it should be continued for three or four weeks. Whether the carbolic acid, without iodine has the same effect, Dr. Rothe does not know. For the last ten years he has used the combination of carbolic acid with iodine in phthisis, diptheria, diarrhœa, etc., and has never ventured to give up its use. Dr. Rothe says he would not have ventured to publish the results of a small number of observations, if it were not for the desire that they should be repeated, and confirmed or corrected. He hopes that his professional colleagues, if they think the treatment worth a trial, will publish the result of their observations.—*London Med. Record*, Aug. 15, 1880.

### PROFESSIONAL APHORISMS.

Would you rid yourself of a bothersome patient? Send him your bill.

The patient who pays his physician is exacting only; the one that does not pay is a despot.

The physician who waits for his fees, trusting to the spontaneous gratitude of his patients, resembles the traveler who waited for the river to stop running so that he could cross.

Exaggerated fees always turn to the confusion of the art and those who practice it. A rich man upon whom a surgeon had just performed a serious operation, received from the latter a demand for an enormous sum. "You ought to have warned me," said the sufferer, "that your way of carrying on your trade is to demand 'your money or your life.'"

When we reflect on the stupid credulity of men in relation to medicine, we ought not to be so much astonished that there are so many charlatans as at the fact that there are still so many honest people among the doctors.

A society lady, noted for her levity, asked her physician how many doctors it would take to make a scholar. He replied, just as many as it would take of lovers to satisfy a coquette.—*St. Louis Clinical Record.*

FRENCH editors are generally very squeamish, and French medical editors have to sacrifice what little delicacy remains in them in the interests of their profession. Hence occurs the following "case" in *Le Practicien*: A young and lovely lady in a railway carriage, with her old and sleepy husband, suffers from toothache. There is a traveling companion, a stranger, eager to make himself agreeable to the young and lovely one while the old man sleeps. He sympathizes with her sufferings, and at last gently insinuates that if he might place his warm lips to her face it might relieve the pain. "No, sir," says the husband, amiably arousing himself, "your remedy is useless for toothache, but it is excellent for piles, from which I suffer."

### ANTISEPTIC SURGERY.

MM. Gosselin and Bergeron are still prosecuting their researches on the mode of action of the antiseptic substances employed in dressing wounds. The question has occurred to them whether the antiseptic action of carbolized alcohol, now largely employed, may not be as well attributed to the alcohol as to the carbolic acid. Having placed each of these substances separately in contact with blood, they have found that the alcoholised blood putrefied less quickly when it was in contact with carbolic acid then when it was mixed with alcohol, and still less quickly in the latter case than when it was pure. The experimenters, therefore, are of opinion than the concurrence of the two substances is useful, the carbolic acid destroying the germs, whilst the alcohol induces coagulation of the blood and consequently its relative imputrescibility.—*British Medical Journal.*

### GRATITUDE OF PATIENTS.

One of Jean Baudry's aphorisms runs thus (*Medical Times and Gazette.*): The gratitude of the patient to his physician! I know that. It is part of the disease. It is declared during the fever, cools down in convalescence, and is cured when health returns.—*The Cincinnati Lancet and Clinic.*

### THE DIPHTHERIA DISCUSSION AT THE MEDICAL SOCIETY.

The subject of Diphtheria has occupied a prominent place in the discussions of the Medical Society during the last three months, and, despite the great variety of opinions expressed, we believe that substantial good has resulted; and this is largely due to the fact that the speakers kept very closely to the detail of their own experience, and did not merely recite the opinions of others, of whose correctness no guarantee could be given. Such a debate is sure to give rise to important results, even though the immediate outcome seem small, for further inquiry is stimulated, and we hope in the near future to receive some valuable papers on this subject from our friends in the country districts.

The bulk of evidence and of argument tended to show that diphtheria is caused by specific poison which infects the system, and, after a certain period of premonitory symptoms, leads to the development in the throat of diagnostic membrane—the anatomical sign or local manifestation of the disease. The main points urged in favour of this view were the close analogy that diphtheria bears to the eruptive fevers in almost every point of its history, the absence of throat lesions in some of the most malignant cases, the occurrence of prodromata in the great mass of instances, and the tendency to albuminuria, and subsequent paralysis.

On the other hand, it was contended by an influential minority that diphtheria is primarily a local affection of the throat, due to the lodgment of germs, which multiply upon the mucous membrane and subsequently poison the whole system, thus producing secondarily the constitutional symptoms and the occasional sequelæ. The supporters of this theory placed most weight upon the results of local treatment, citing numerous cases in which well-marked attacks were cut short in the early stage by the topical use of disinfectants or caustics. Stress was also laid upon the history of the diphtheritic membranes that form on wounds, and on the analogy that the disease bears to erysipelas, septicæmia, and other affections which may occur repeatedly in the same individual, and *may possibly* be independent of any pre-existing case.

We trust that one result of this debate will be that diptheria will be clearly distinguished in future from the less serious lesions of the throat, which hitherto have been so commonly confounded with it; and then it may be confidently expected that further observations will soon lead to decisive results in unravelling the tangled web of facts and false facts which has involved all reasonings concerning the origin and treatment of this so fatal a disease.—*Australian Medical Journal.*

### PLEURITIC EFFUSION—TREATMENT BY HYPODERMIC INJECTIONS OF PILOCARPIN.

Dr. Haminio Tassi (*Jour. des. Sci. Med.*; from the Italian) found, in a case of pleuritic effusion on the left side, that the greatest relief was given by injections of pilocarpin in the dose of one-third to one-half grain daily, which were well borne. The following symptoms were observed. The pulse increased in frequency a little after the injection, and continued thus for a short time. The temperature likewise increased within about six minutes, continuing so for some twenty minutes. At this time perspiration was established, after which the temperature gradually diminished to a degree somewhat lower than before the injection. Sialorrhœa showed itself a few minutes after the injection, and lasted several hours. There was no hiccough, nor any vomiting; but secretion of tears preceded perspiration, and sometimes lasted about an hour, and was confined to the right eye. Pupillary atresia was observed in only a single case; there was always dilatation; urine was scanty.—*Philadelphia Medical Times.*

### SIMPLE METHOD OF REDUCING PARAPHIMOSIS.

M. Bardinet employs the following with success. He inserts the convex ends of three hair pins, at regular distances apart, beneath the constricting ring, and over the bridge thus formed the foreskin is drawn down with the greatest facility.—*Alleg. Med. Cent. Zeit.*

### COMPRESSION OF THE FEET OF CHINESE WOMEN.

Specimens of Chinese ladies' feet are tolerably familiar, preserved in museums; and the wasted condition of the tissues of the leg and foot has been often described. But the manner in which the deformity is produced, and the tortures to which girls are subjected, that their feet may be "quite in the fashion," are not, perhaps, so familiar to English readers. The process is thus graphically described by an American missionary, Miss Norwood, of Swatow. She says that the binding of the feet is not begun till the child has learnt to walk and do various things. The bandages are specially manufactured, and are about two inches wide and two yards long for the first year, five yards long for subsequent years. The end of the slip is laid on the inside of the foot, at the instep, then carried over the toes, under the foot, and round the heel, the toes being thus drawn towards and over the sole, while a bulge is produced on the instep, and a deep indentation, in the sole. The indentation, it is considered, should measure about an inch and a-half from the part of the foot that rests on the ground up to the instep. Successive layers of bandages are used till the strip is all used, and the end is then sewn tightly down. The foot is so squeezed upward that, in walking, only the ball of the great toe touches the ground. Large quantities of powdered alum are used to prevent ulceration and lessen the offensive odor. After a month, the foot is put in hot water to soak some time; then the bandage is carefully unwound, much dead cuticle coming off with it. Ulcers and other sores are often found on the foot; frequently, too, a large piece of flesh sloughs off the sole, and one or two toes may even drop off, in which case the woman feels afterwards repaid by having smaller and more delicate feet. Each time the bandage is taken off, the foot is kneaded, to make the joints more flexible, and is then bound up again as quickly as possible with a fresh bandage, which is drawn more tightly. During the first year the pain is so intense that the sufferer can do nothing, and for about two years the foot aches continually, and is the seat of pain which is like the pricking of sharp needles. With continued rigorous binding, the foot in two years becomes dead and ceases to ache, and the whole leg, from the knee downward, becomes shrunk, so as to be little more than skin and bone. When once formed, the "golden lily," as the Chinese lady calls her delicate little foot, can never recover its original shape.—*Brit. Med. Journal.*

### THE PHYSICIAN IN THE SCHOOL-ROOM.

It is a matter of congratulation whenever a respectable medical man is placed upon a school board, for there is then a possibility that the health of the children, during school hours, will not be neglected. In this we of course assume that the physician occupying such an official position possesses an average amount of common sense, superadded to the fair share of information on hygiene which has been acquired by him during attendance on an educational course at some respectable medical school. The number of medical men upon the various school boards throughout the country is by far too limited, and the vital questions of human health are frequently left in the hands of political demagogues and tricksters, whose whole attention is devoted to matters very remote from hygienic considerations. The greater amount of interest now taken in the preservation of the health of school children is almost entirely due to the influence, in this direction, exerted by the profession, collectively and individually, in and out of educational boards.

It would be eminently proper that representatives of the medical profession should, in every town in the land, bring to bear upon all school boards the pressure of the present advanced views on hygiene that have been so long neglected in the construction and equipment of rooms devoted to the instruction of the young. For the sake of these future citizens themselves, and of their happiness in all their domestic and social relations in the years to come, let the profession persevere unceasingly in this good work, searching for violations that already exist of hygienic laws, recommending such changes as may be beneficial, and insisting, to the very end, upon the uprooting of every radical cause of disease and death in schools of instruction. Let them give to the tender plants, which others may neglect, the air and sunshine which they absolutely need for their preservation and development.—*The College and Clinical Record.*

### ATROPIA AGAINST CARDIO-INHIBITORY EFFECTS OF CHLOROFORM.

In the *British Medical Journal,* October 16, 1880, by Mr. E. A. Schafer, F. R. S., of the Physiological Laboratory of the University College, London, is a notice of an inquest upon the body of a person who died during the administration of chloroform after the heart's action had suddenly stopped; besides the employment of artificial respiration, the other means taken to effect resuscitation was the hypodermic injection of atropin and the application of the galvanic battery to the cardiac region. Mr. Schafer says now, since it is well known that atropin paralyzes the cardiac inhibitory apparatus, there undoubtedly seems good reason for the employment of atropin. But clearly it should always be given immediately before the administration of chloroform as a preventive, for if the heart's action has completely stopped, the circulation having once ceased, of course no cure is possible; and even if the inhibitory actions have only much slowed and weakened the heart without having actually arrested it, the absorption of the atropin would probably be too long delayed to be of any avail. With regard to the other remedy—the application of the galvanic current to the cardiac region—the effect of direct stimulation of the heart is so opposite, that it is no exaggeration to say that the treatment is at least as likely to arrest a beating heart as to set an inhibited one in activity.—*The Medical Herald.*

### TREATMENT OF PULMONARY PHTHISIS BY BENZOATE OF SODIUM.

In the reports of Dr. Augusto Murri (*Rivista Clinica di Bologna,* 1880, p. 24) the following appears:—

When, from the service of Rokitansky, came the consoling news of the cure of phthisis by benzoate of sodium, the author commenced in his wards its administration, both by inhalation (following Rokitansky's rules) and internally, to combat the supposed specific poison which tends to invade the entire organism.

But it was found impossible, in using the fi.e per cent. solution, to administer to any patient the large daily dose of 50 grams recommended by Rokitansky (*Wiener Medicin. Presse*, No. 3742, 1879) or even the smaller dose given by Schuler (*Berliner Klinisshe Wochenschrift*, No. 45, 1879).

The largest dose it was found possible to administer was sixteen grams; it is, in fact, impossible to make a sufficient number of inspirations to consume these large doses; after inhaling a short time nausea and vomiting renders its further use for the moment intolerable to the patient. Again, it is impossible to administer this large dose internally, for it induces a sensation of burning at the stomach, with great pain in the abdomen and diarrhœa; this is what occurred in the case of a patient determined, if possible, to follow out the treatment, and obliged to give it up on account of the vomiting and abdominal pains it induced; it is true, these troubles cease after a time, but it requires a patient of exceptionally firm resolution to follow the treatment thus far.

This intolerance of the remedy by the patients would not cause its banishment from therapeutics if it gave advantageous results; but in twelve cases of phthisis, where the treatment was continued more than two months, the progress of the disease was not arrested; in fact caverns were formed where none before existed, and several of the patients died while under treatment.

Again, it was affirmed by Rokitansky, that the fever ceded immediately, but it was found to continue its irregular course, with, perhaps, jus a slight remission at the beginning of the treatment.

The sole advantage seeming to result from the use of the remedy, but that during the first few days only, was greater facility of expectoration and lessening of the cough; but how many remedies less disagreeable and less dangerous have similar virtues.

These experiences in the Bologna Clinic do not seem encouraging enough to introduce the general use of this remedy, so much vaunted by Rokitansky in the treatment of phthisis.—*Med. and Surg. Reporter.*

### ANÆSTHETICS IN LABOR.

Dr. Herriott, in his report on Obstetrics to the Illinois State Medical Society, published in the St. Louis *Medical and Surgical Journal*, October 5th, 1880, says:

The indications for the use of anæsthetics are undue suffering, such as from a rigid os, tention of the soft parts, or irregular contractions of the uterus. Get the patient under mental control and put her gradually but fully under the influence of the anæsthetics for twenty to thirty minutes. Malposition can be corrected during this time. If the patient is not in the act of expulsion allow her to return to semi-consciousness. I have, in three-quarters of an hour, delivered women who would have had to wait from four to six hours.

In primipara the apprehension of evil adds to the nervous exhaustion and the cervix does not dilate as readily.

The anæsthetic quiets undue nervous excitement, husbands the strength, restrains reflex, irregular and excessive uterine contractions. The normal uterine action is increased and not diminished under its influence.

In what special cases are we to refrain from the use? I answer in no case, unless no benefit can be obtained. I have used twenty grains chloral hydrate and followed with chloroform.

In a letter, Dr. Darrah, a member of this society, says that anæsthetics have a moral effect in encouraging subsequent pregnancies. He would not use them in cases of inertia and anæmia, where no special demand for them existed.

Dr. Wheeler Jones, our Vice-President, wrote to me that he had used anæsthetics in labor twelve years. He notes especially the moral effect, but refrains from the use in the first stage, except if eclampsia exists; he also refrains from the use in cases of organic disease of the heart.

Scrutinize your patients and exercise care, and scarcely a case will be found in which organic disease does not prevent gestation to full term. Anæsthetics in such cases as arrive to full term can and ought to be given. So in regard to hemorrhage, it ought not to deter us unless absolute danger exists.

I had a case where there was great suffering, and twice, previously, parturition had occupied from sixteen to seventeen hours. I gave chloroform and the labor occupied four and a-half hours; lasting two and a-half hours after administering the anæsthetic. In fifteen minutes after birth the placenta was expelled and less than the usual amount of hemorrhage ensued.

The time to commence the use of an anæsthetic is when the patient without it would suffer; and the time to withdraw it, is when the patien twithout it would not suffer.—*Med. and Surg. Reporter.*

### IS SHAMPOOING ADVISABLE?

Dr. Rumbold, in his book on catarrh, says no! He writes of it: This is injurious to the scalp and the hair; it removes every particle of oil from the head, causing the scalp to become dry and full of dandruff, and the hair to lose its glossiness and natural color, generally giving it a faded and lighter appearance; but worse than this, on account of the absence of the oil, the patient is more liable to take cold, on even a slight exposure of head to a draught of cool air.

Dr Leonard, on the other hand, in his book on the hair, recommends that it be washed several times a week with soap and water.

Let us have this question brought to an issue and settled.—*Med. and Surg. Repoıter.*

### THE MORTALITY OF PHYSICIANS.

Dating from the horizon of South Germany, Prof. Von Hecker, of Munich, finds that in the first ten years of their professional life (twenty-four to thirty-four) about six per cent. of physicians die; while in the next decade (thirty-four to forty-four) more than twelve per cent. meet their end. He attributes this to exhaustion from excessive labor, neglect of slight ailments, and also to the abuse of alcohol and opium (morphine), which latter he believes is more common among the members of the medical than any other profession. *Med. and Surg. Reporter.*

INJECTION of nasal polypi with a fifty per cent. dilution of acetic acid, has been successfully practiced at the Louisville Eye and Ear Infirmary, and at the Hospital College Clinic.—*Louisville Medical Herald.*

### OCT'R REPORT OF DEATHS AND INTERMENTS IN BALTIMORE.

| CAUSE OF DEATH. | No. | CAUSE OF DEATH. | No. | CAUSE OF DEATH. | No. |
|---|---|---|---|---|---|
| Abscess, Psoas | 1 | Delirium Tremens | 1 | Metro Peritonitis | 1 |
| Anæmia (Pernicious) | 1 | Diarrhœa | 10 | Malig't Disease of Stomach | 1 |
| Aneurism of Aorta | 1 | Diphtheria | 37 | Meningitis | 15 |
| Aortic Incompetency | 1 | Disease of Brain | 1 | " Tubercular | 4 |
| Albuminaria | 1 | " Heart | 27 | " Cerebro Spinal | 3 |
| Apoplexy | 7 | Dropsy, (Gen'l) | 5 | Murder | 2 |
| Acute Yellow Atrophy Liver | 1 | " Scarlatinal | 2 | Malarial Toxaemia | 1 |
| Aortic Reguretation | 1 | Drowned | 3 | Nervous Prostration | 1 |
| Asphyxia | 1 | Dysentery | 4 | Old Age | 22 |
| Asthma | 5 | Emphysema | 1 | Paralysis | 13 |
| Asthenia | 9 | Epilepsy | 1 | Pneumonia | 33 |
| Atrophy | 1 | Exzema | 1 | " Typhoid | 3 |
| Bright's Disease | 7 | Erysipelas | 1 | " Broncho | 1 |
| Burns | 2 | Epithehems of Axilla | 1 | " Pleuro | 1 |
| Capillary Bronchitis | 4 | Fever, Gastric | 1 | Poison | 3 |
| Cancer, Neck | 1 | " Intermittent | 5 | Premature Birth | 17 |
| " Abdominal Cavity | 1 | " Malarial | 7 | Protracted Labor | 1 |
| " Breast | 1 | " Puerperal | 3 | Purpura Hemorrhagica | 1 |
| " Bowels | 1 | " Scarlet | 52 | Puerpural Peritonitis | 1 |
| " Liver | 1 | " Typhoid | 28 | Placentia Praevia | 2 |
| " Nasal Cav.& Orbits | 1 | " Typhus | 2 | Phthisis Laryngeal | 1 |
| " Oesophagus | 1 | " Typho-Malarial | 3 | Rheumatism | 1 |
| " Rectum | 1 | Gastro Enteritis | 2 | " Heart | 1 |
| " Peritoneum | 1 | Gout | 1 | Scrofula | 3 |
| " Uterus | 3 | Hernia, Strangulated | 2 | Suppression of Menses | 1 |
| " Stomach | 6 | Hemorrhage, Bowels | 2 | Septicæmia (Puerperal) | 1 |
| Calculus | 1 | " Umbilical | 3 | Selerosis of Liver | 1 |
| Child Birth | 2 | Hydrocephalus | 3 | Senile Catarrh | 1 |
| Cholera Infantum | 7 | Hydro Percardium | 1 | Suicide | 2 |
| " Morbus | 1 | Inanition | 10 | Syphilis | 3 |
| Cirrhosis of Liver | 3 | Inflammation of Brain | 1 | Thrombosis | 1 |
| Concussion of Brain | 3 | " Bladder | 4 | Tetanus | 2 |
| Congestion of Brain | 2 | " Stomach | 4 | Trismus Nascentium | 6 |
| " Liver | 1 | " Bronchi | 6 | Traumatic Peritonitis | 1 |
| " Lungs | 3 | " Kidneys | 1 | Uræmia | 2 |
| " Bowels | 1 | " Pericard'm | 1 | Unknown, Adult | 1 |
| Consumption of Bowels | 1 | " Peritoneum | 5 | Unknown Infantile | 4 |
| " Lungs | 112 | " Tonsils | 1 | Whooping Cough | 22 |
| Convulsions | 36 | Intemperance | 7 | Wounds | 4 |
| Croup | 25 | Malformation | 2 | Starvation, ill treatment | 1 |
| Debility | 1 | Mal-nutrition | 3 | | |
| Dentition | 11 | Marasmus | 24 | Total | 735 |

THE DEATHS REGISTERED IN THE MONTH INCLUDE

| | | | |
|---|---|---|---|
| Under 1 year | 182 | Between 20 and 30 " | 77 |
| Between 1 and 2 years | 63 | " 30 and 40 " | 63 |
| " 2 and 5 years | 58 | " 40 and 50 " | 51 |
| | | " 50 and 60 " | 37 |
| Under 5 years | 303 | " 60 and 70 " | 52 |
| Between 5 and 10 years | 69 | " 70 and 80 " | 34 |
| " 10 and 15 " | 8 | " 80 and 90 " | 22 |
| " 15 and 20 " | 18 | " 90 and 100 " | 1 |

NATIVITY, ETC.

| | |
|---|---|
| United States—White | 463 |
| " Colored | 158 |
| Foreign | 114 |
| Still Birth | 53 |
| Total for the month | 735 |

Among the decedents were 61 above the age of 70 years, whose combined ages were 4,811 years, averaging 78 years 10 months.

Estimated Population, 393,796. White, 338,384. Colored, 55,412. Annual Death Rate during the month, whole population, per 1,000—22.39. White, 20.48. Colored, 34.47.

By Order: JAMES A. STEUART, M. D., Commissioner of Health and Registrar.

A. R. CARTER, Secretary.

U. S. SIGNAL OFFICE, BALTIMORE, MD., November 1, 1880.

### METEOROLOGICAL SUMMARY FOR THE MONTH OF OCTOBER, 1880.

### PRESSURE.

| | | |
|---|---|---|
| Mean Monthly Barometer, | 30.139 inches. | |
| " " " for past 10 years, | 30.098 " | |
| Highest Barometer, | 30.503 " | on the 19th. |
| Lowest " | 29.508 " | on the 30th. |
| Monthly Range of Barometer, | .995 " | |

### TEMPERATURE.

Mean Monthly Thermometer, - - - - - 56.0 degrees.
" " " for past 10 years, - 57.4 "

| | | | |
|---|---|---|---|
| Highest Thermometer, | 81 degrees on 16th. | Monthly Range, | 46 degrees. |
| Lowest " | 35 " on 19th. | Mean Daily Range, | 16.8 " |

### WIND, ETC.

| | |
|---|---|
| Prevailing Direction of Wind, Southeast. | Total Amount of Rainfall, 2.64 inches. |
| MeanVelocity of Wind, 5.3 miles per hour. | Number of Days on which Rain fell, 12. |
| Highest Velocity of Wind, 24 " " on 23d. | Mean Relative Humidity, 66.8 per cent. |
| Total Movement of Wind, 3,939 miles. | ROBERT SEYBOTH, in charge. |

